# Non-neuronal cholinergic activity is potentiated in myasthenia gravis

**DOI:** 10.1186/s12883-016-0772-3

**Published:** 2017-02-08

**Authors:** Bin Han, Chao Zhang, Shoufeng Liu, Yiping Xia, Hao Sun, Zhongying Gong, Alain R. Simard, Qiang Liu, Junwei Hao

**Affiliations:** 1 Department of Neurology, Tianjin Neurological Institute, Tianjin Medical University General Hospital, Tianjin, 300052 China; 2 Department of Neurology, Tianjin HuanHu Hospital, Tianjin, 300060 China; 3 Laboratory of Physical and Chemical Research, Tianjin Centers for Disease Control and Prevention, Tianjin, 300011 China; 4 Department of Neurology, Shandong Provincial Hospital Affiliated to Shandong University, Jinan, Shandong 250021 China; 5 Department of Neurology, Tianjin First Center Hospital, Tianjin Medical University, Tianjin, 300192 China; 6 Département de chimie et biochimie, Université de Moncton, Moncton, New Brunswick E1A 3E9 Canada; 7 Department of Neurology, Barrow Neurological Institute, St. Joseph’s Hospital and Medical Center, Phoenix, AZ 85013 USA

**Keywords:** Acetylcholine, Myasthenia gravis, Inflammation, Cholinergic anti-inflammatory pathway

## Abstract

**Background:**

Non–neuronal acetylcholine (ACh) restricts autoimmune responses and attenuates inflammation by cholinergic anti-inflammation pathway. To date, the implication of ACh in myasthenia gravis (MG) remained unexplored. This study aimed to investigate the possible relationship between ACh levels, anti–muscle-specific tyrosine kinase (MuSK) antibody titers, main clinical features and outcomes of MG patients.

**Methods:**

We successfully measured ACh levels in human peripheral blood mononuclear cells (PBMCs) from 125 MG patients and 50 matched healthy controls by using ultra-performance liquid chromatography-tandem mass spectrometry (UPLC-MS/MS). We assessed the quantitative MG (QMG) scores for each patient and titered anti-MuSK antibody.

**Results:**

We found that PBMC-derived ACh level was significantly higher in MG patients, especially in patients of class III, IV-V, compared with that in controls (0.142 ± 0.108 vs. 0.075 ± 0.014 ng/million cells, *p* = 0.0003) according to the Myasthenia Gravis Foundation of America clinical classification. Importantly, we also found that ACh levels were positively correlated with QMG scores (*r* = 0.83, *p* < 0.0001) and anti–MuSK Ab levels (*r* = 0.85, *p* < 0.0001).

**Conclusions:**

Our demonstration of elevated ACh levels in PBMCs of MG patients foreshadows potential new avenues for MG research and treatment.

**Electronic supplementary material:**

The online version of this article (doi:10.1186/s12883-016-0772-3) contains supplementary material, which is available to authorized users.

## Background

Myasthenia gravis (MG) is a well-recognized autoimmune disorder affecting neuromuscular junction [1, 2]. Inflammatory environment plays a crucial role in the progress of this disease [3], appropriate interference with inflammation is a very promising therapeutic strategy and requires further research.

Recently, studies regarding the immune-modulating properties of acetylcholine (ACh) derived from non-neuronal cells has prompted an increasing interest in the field of the cholinergic anti-inflammatory pathway. Interestingly, non-neuronal ACh and main components of the cholinergic system, such as choline acetyltransferase (ChAT), acetylcholinesterase (AChE), vesicular acetylcholine transporter (VAChT) and high-affinity choline transporter (ChT1), are expressed in multiple human cell types, including keratinocytes [4], pancreatic cells [5] and immune cells [6]. For instance, ACh released by ChAT^+^ B cells limits local neutrophil recruitment to modulate innate immunity during sterile endotoxemia [7]. Additionally, ACh-producing T cells in the spleen, induced by vagus nerve stimulation, are known to decrease the intensity of inflammatory responses through the inhibition of TNF–α production [8]. Lymphocyte-derived ACh may thus play a key role in the interaction between the nervous system and immune system to restore homeostasis, a mechanism called the ‘cholinergic anti-inflammatory pathway’ [9, 10]. Targeting this pathway, which reduces pro-inflammatory cytokine secretion, was effective in many experimental animal models, including inflammatory bowel disease [11], experimental autoimmune encephalomyelitis (EAE) [12–16], arthritis [17], ischemia-reperfusion injury [18], sepsis [19, 20], pancreatitis [21], myocardial ischemia [22] and hemorrhagic shock [23]. These studies suggest a tight inter-relationship between non-neuronal ACh and inflammatory reactions. However, peripheral cholinergic function related to ACh has not been investigated in MG till now.

These observations brought us to question whether human PBMC-derived ACh play a critical role in the process of MG. Here, we described a simple and stable ultra-performance liquid chromatography-tandem mass spectrometry (UPLC-MS/MS) method of our design for quantifying ACh levels in peripheral blood mononuclear cells (PBMCs). In addition, we investigated the relationships between ACh levels and MG severity.

## Methods

### Subjects

This study was approved by institutional review boards from Tianjin Medical University General Hospital, Tianjin Huanhu Hospital, Shandong Provincial Hospital Affiliated to Shandong University and Tianjin First Center Hospital. Informed consent was obtained from all participants prior to inclusion. For this study, 125 eligible patients diagnosed with MG for the first time and 50 matched healthy individuals were enrolled from April 2015 to May 2016. The optimum diagnosis of MG was based on a combination of patients’ clinical manifestations, abnormal electrodiagnostic studies on single-fiber electromyography testing, abnormal repetitive nerve stimulation and previous response to treatment. Aberrant levels of anti-acetylcholine receptor (AChR) and anti–muscle-specific tyrosine kinase (MuSK) antibodies supported the diagnosis; however, a seronegative antibody titer was considered insufficient to refute the diagnosis of MG. All the patients received no cholinesterase inhibitors and immunosuppressive agents before definite diagnosis of MG. Exclusion criteria included patients with cardiovascular and cerebrovascular diseases, coagulation disorders, pregnancy, other disorders causing weakness or fatigue, combined with other autoimmune diseases, history of malignant disease except for thymus, inability to provide informed consent. MG subtyping followed the Myasthenia Gravis Foundation of America (MGFA) clinical classification, and the severity of disease and effectiveness of treatment were assessed by a validated quantitative MG Scoring system (QMG Score) [24].

### Isolation of human PBMCs

Before patients receiving treatment, venous blood samples were extracted into ice-cold tubes containing EDTA and centrifuged immediately thereafter at 3000 rpm for 10 min, separating the samples into two layers. The upper layer, plasma, was subpackaged and stored at -80 °C for further analysis. The lower layer, precipitated blood cells, was the source of PBMCs isolated with Ficoll density gradients.

### Detection of PBMC-derived intracellular ACh by UPLC–MS/MS

A stable method developed by our team was used to detect ACh levels [25]. Briefly, the fresh PBMCs were separated from blood and added to 100 μL deionized H_2_O/0.1% formic acid (vol:vol), and vortexed for 15 s to make homogenate, which was de-proteined by adding 300 μL acetonitrile containing isotope internal standard (D9-ACh, C/D/N, Quebec, Canada). The sample was vortexed again for 1 min then centrifuged at 15000 rpm for 10 min. The supernatant was transferred to a sterile autosampler glass vials for measurement. The recovery of ACh following the sample cleanup procedure, as estimated from the pre and post cleanup standard curves, was 96.5%.

For highly specific and sensitive measurement of intracellular ACh, we used an Acquity UPLC system coupled to a Xevo TQ-S (Waters Corporation, Milford, MA, USA). ACh was separated by liquid chromatography using a CORTECS HILIC column (1.7 μm, 100 mm × 2.1 mm). Serially diluted ACh was used to construct a standard curve. All standards and samples received a uniform aliquot of D9-ACh internal standard to correct for variations in extraction efficiency.

### Anti-AChR and anti–MuSK Ab titering

Anti-AChR and anti–MuSK Ab levels in the blood plasma were determined in duplicates, and mean values calculated, by ELISA (Trust Specialty Zeal, USA [AChR Ab assay range: 6 pmol/L-1200 pmol/L; MuSK Ab assay range: 2 U/L-400 U/L]) according to the manufacturer’s instructions.

### Quantitative real-time PCR (qRT-PCR)

Total RNA was extracted from fresh human PBMCs with the TRIzol reagent (Invitrogen, USA) according to the manufacturer’s protocol. Reverse transcription reactions were carried out with TransScript First-Strand cDNA Synthesis Super Mix (TransGen Biotech, China) according to the supplied protocol. qPCR reactions were all performed in triplicate. FastStart Universal SYBR Green Master (Roche, Germany) was used as the detection dye. We used β-actin as an internal reference gene to normalize the expression level for AChE, ChAT, ChT1 and VAChT. The amplification and detection of specific products were performed with the CFX Connect^TM^ Real-Time System (Bio-Rad, USA). PCR conditions were the following: denaturation at 95 °C for 10 min, followed by 39 cycles of 95 °C for 15 s, 58-61 °C for 30 s, 72 °C for 20 s, and a final extension step of 72 °C for 10 min. The mean value was calculated by plotting Ct, and then used for further calculation. The mean relative expression was gained by the 2^-△△Ct^ comparative method. The primers for amplification were the following: 5′-GGGTGGTAGACGCTACAACC-3′ (forward) and 5′-GTGCCCTCAAAACCTGGGTAT-3′ (reverse) for AChE, 5′- AACCACGGAGATGTTCTGCTGCTAT-3′ (forward) and 5′- TTGTTGCCAATGGCTTGCTCTCAG-3′ (reverse) for ChAT, 5′-ATCCCAGCCATACTCATTGG-3′ (forward) and 5′-CAGAAACTGCACCAAGACCA-3′ (reverse) for ChT1, 5′- GGCATAGCCCTAGTCGACAC-3′ (forward) and 5′-CGTAGGCCACCGAATAGGAG-3′ (reverse) for VAChT.

### Western blot analysis

Human PBMCs were lysed in RIPA (Solarbio) coupled with PMSF, a protease inhibitor. Afterward, we detected protein concentrations using BCA protein assay reagent; the equal protein samples were then mixed with 4 × sample buffer and boiled for 10 min. The samples were separated by 10% SDS-PAGE gel and transferred onto PVDF membranes (Millipore, USA) by the semi-dry transfer method. After blocking in 5% nonfat dried milk in TBST for 2 h at room temperature, the blots were incubated with specific antibodies overnight at 4 °C. After washing, the membranes were incubated with secondary antibodies for 1 h at room temperature. The specific protein bands were detected using a Bio-Rad Gel Doc Imager. The specific antibodies (Santa Cruz, CA, USA) included were mouse anti-AChE (1:100), mouse anti-ChAT (1:100) and goat anti-VAChT (1:1000). The relative amounts of proteins were normalized against GAPDH.

### Determination of cholinergic components in PBMCs by ELISA

Human PBMCs were lysed, and total protein concentration of the lysates was determined by BCA method. The levels of AChE, VAChT and ChAT proteins were then measured by ELISA (Shanghai Yuanye biological technology co., LTD, Shanghai, China) according to the manufacturers’ instructions. All samples and standards were determined in duplicate. All data were calculated and normalized to the total protein concentration in each sample.

### Statistical analysis

Statistical analysis was performed using SPSS Statistics 20.0 (IBM Corp, USA. Released 2011). Data are expressed as means ± SD. For data with normal distribution, one-way analysis of variance with Dunnet test was used to assess the differences among groups. An independent-sample *t* test was used to compare differences between two groups. For data with nonnormal distribution, the Mann–Whitney *U* tests to compare differences between two groups. Kruskal-Wallis test was applied to test for mutiple groups. Repeated measures ANOVA was used to compare differences before and after treatment. Regression analysis was conducted to test for associations between parameters (ACh, QMG scores and anti–MuSK Ab concentrations). Values of *p <* 0.05 were considered to be statistically significant.

## Results

### Baseline characteristics

The characteristics of all participants enrolled in this study were shown in Table 1. There were no statistically significant differences in age, or gender in the MG patients as compared with healthy controls. In addition, we have shown the treatment plan after blood drawing in this table.

**Table 1:** Comparative demographics of patients with MG and controls

Characteristics	MGFA Classification				Control
	I	II	III	IV-V	
NO. of samples(n)	58	30	24	13	50
Age (years)	46.10 ± 9.45	43.80 ± 10.83	45.63 ± 10.94	48.23 ± 9.65	46.14 ± 9.89
Female n (%)	28 (48.3)	15 (50.0)	10 (54.2)	5 (45.5)	26 (52.0)
AChR Ab pos n (%)	42 (72.4)	20 (66.7)	17 (70.8)	7 (53.8)	NA
MuSK Ab pos n (%)	3 (5.2)	3 (10.0)	4 (16.7)	5 (38.5)	NA
Double negative n (%)	13 (22.4)	7 (23.3)	3 (12.5)	1 (7.7)	
Treatment	Pyridostigmine, 23 of 58 added oral Prednisone	Pyridostigmine, 25 of 28 added oral Prednisone	Pyridostigmine, IVIG, Subsequent oral prednisone	Pyridostigmine, IVIG, IV methylprednisolone	NA

*MG* myasthenia gravis, *AChR Ab* acetylcholine receptor antibody, *MuSK Ab* muscle-specific tyrosine kinase antibody, I to V according to Myasthenia Gravis Foundation of America (MGFA) clinical classification; *NA* not available

### PBMC-derived ACh content changed in MG patients compared with controls

**Fig. 1:**
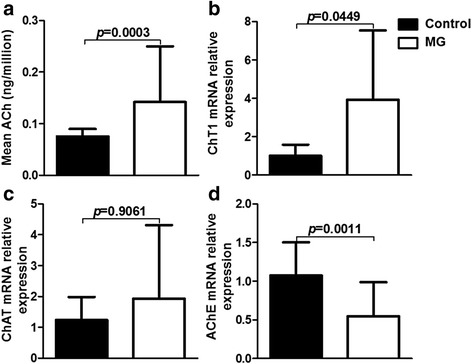
PBMC-derived ACh and cholinergic content differs in patients with MG from that in healthy controls. **a** PBMC-derived ACh per million cells was obviously increased in MG patients (*n* = 125) compared with controls (*n* = 50) (*p* = 0.0003). **b** High-affinity choline transporter (ChT1) mRNA expression was higher in MG patients (*n* = 15) than that in control (*n* = 6) (*p* = 0.0449). **c** Choline acetyltransferase (ChAT) mRNA relative expression showed no obvious change (*p* = 0.9061). **d** A sharp decrease in acetylcholinesterase (AChE) mRNA expression was observed between MG patients and controls (*p* = 0.0011). (**c-d**; *n* = 10 for control, *n* = 36 for MG)

We first established that UPLC-MS/MS was a precise, stable and available technique to detect intracellular ACh then used this method routinely to determine the PBMC-derived ACh content of our MG patients. To eliminate the influence of cholinesterase inhibitors, we assessed ACh levels by preprocessing PBMCs with eserine, a cholinesterase inhibitor, for 30 min in the vacuette blood collection tubes once blood samples were drawn and compared MG patients untreated or treated with pyridostigmine (Additional file 1). We did not find statistically significant differences (*p* > 0.05). Eight patients were in each group, and the two groups showed no significant differences in QMG score (2.00 ± 1.604 vs. 1.75 ± 1.488, *p* = 0.7513). Subsequently, we recorded significantly higher levels of ACh per million cells in MG patients compared with controls (0.142 ± 0.108 vs. 0.075 ± 0.014 ng/million cells, *p* = 0.0003) (Fig. 1a). Similarly, amount of relative mRNA expression for ChT1, the rate-limiting enzyme for ACh, was higher in samples from MG patients than those from controls (*p* = 0.0449) (Fig. 1b). However, there was no statistical significance for ChAT, a direct synthetase for ACh, mRNA expression (*p* = 0.9061) (Fig. 1c). The MG group contained dramatically less AChE, a degrading enzyme for ACh, mRNA than in controls (*p* = 0.0011) (Fig. 1d).

### Intracellular ACh levels in relation to MG severity

**Fig. 2:**
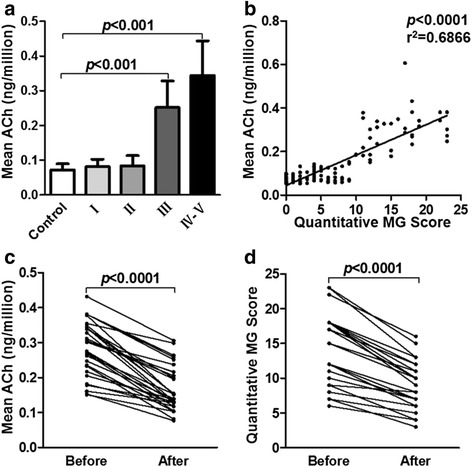
PBMC-derived ACh levels correlate positively with MG severity. **a** Histogram shows that ACh concentrations per million cells increased along with MG severity according to the MGFA clinical classification (*n* = 50 for control, *n* = 58 for class I, *n* = 30 for class II, *n* = 24 for class III, *n* = 13 for class IV-V) (*p* < 0.0001). **b** ACh level distribution relative to QMG scores (*n* = 125) (*p* < 0.0001). **c** ACh content decreased in patient’s blood drawn before treatment and 2 weeks after initial treatment, respectively (*p* < 0.0001). **d** QMG scores also decreased in patients (*p* < 0.0001). (**c-d**; *n* = 30 in each group)

**Fig. 3:**
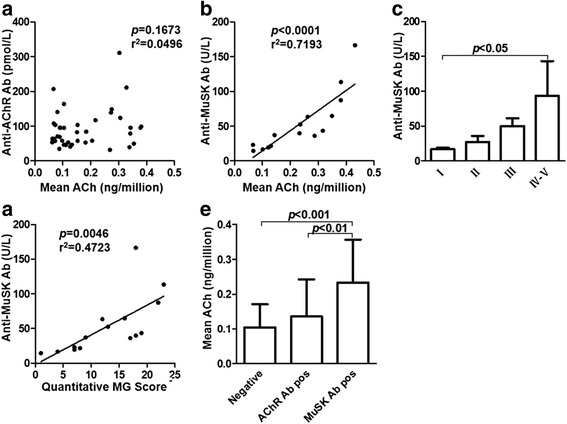
PBMC-derived ACh is positively associated with anti-MuSK Ab concentrations. **a** No correlation was found between ACh levels and anti-AChR antibodies (*n* = 40) (*p* = 0.1673). **b** A positive correlation was found between ACh levels and anti-MuSK antibodies (*n* = 15) (*p* < 0.0001 by regression analysis). **c** Histogram shows that anti-MuSK Ab concentrations relative to MG severity (*n* = 3 for class I and II, *n* = 4 for class III, *n* = 5 for class IV-V) (*p* = 0.0135 by Kruskal-Wallis). **d** Anti-MuSK Ab distributions in relation to QMG scores (*n* = 15) (*p* = 0.0046 by regression analysis). **e** The ACh levels were higher in anti-MuSK Ab-positive group than that in double-negative and anti-AChR Ab-positive groups (*n* = 24 in double-negative group, *n* = 86 in anti-AChR Ab-positive group, *n* = 15 in anti-MuSK Ab-positive group) (*p* < 0.001, *p* < 0.01, respectively)

Based on these findings, we further defined whether ACh levels differ in patients with varied subtypes of MG as classified by the MGFA. ACh content was significantly elevated in patients with class III-V MG (*p* < 0.0001), but not those in class I-II, compared with controls (Fig. 2a). In support, a positive correlation was found between ACh levels and QMG scores in our cohort (*r* = 0.83, *p* < 0.0001) (Fig. 2b). Meanwhile, correlation between MGFA classification and the QMG score in MG patients (*n* = 125) was assessed using the Spearman rank correlation analysis, and positive correlation was observed between them (*r* = 0.874, *p* < 0.001). Also, we observed that ACh levels had declined significantly in patients two weeks after the initial treatment (0.286 ± 0.077 vs. 0.167 ± 0.064, *p* < 0.0001) (Fig. 2c). Eighteen of these patients were treated with pyridostigmine, IVIG and oral prednisone, while others were treated with pyridostigmine, IVIG and IV methylprednisolone. Further, disease severity evaluated by QMG scores before and after treatment with immunological therapy decreased at the post-treatment period to the level of statistical significance (14.20 ± 5.176 vs. 9.07 ± 3.562, *p* < 0.0001) (Fig. 2d). Therefore, our data showing that immunological therapy was effective treatment is consistent with previous studies [26, 27]. We also quantified the anti-AChR and anti–MuSK Ab levels in the plasma of MG patients. No significant association was found between anti-AChR Ab levels and ACh levels (*r* = 0.22, *p* = 0.1673) (Fig. 3a), however, the ACh levels correlated positively with anti–MuSK Ab levels (*r* = 0.85, *p* < 0.0001 by regression analysis) (Fig. 3b). In our cohort, the anti–MuSK Ab concentrations increased as MG worsened (*p* = 0.0135 by Kruskal-Wallis) (Fig. 3c). Meanwhile, a positive correlation was found between the anti–MuSK Ab levels and QMG scores (*r* = 0.69, *p* = 0.0046 by regression analysis) (Fig. 3d). In addition, we found that the ACh levels are significantly elevated in anti-MuSK Ab positive group compared with double-negative and anti-AChR Ab-positive groups (*p* < 0.001, *p* < 0.01, respectively). However, there was no significant statistical differ ence between double-negative and anti-AChR Ab-positive groups (*p* > 0.05) (Fig. 3e). Overall, some of our findings are in accord with those from previous studies [28–30]. The increased ACh levels denoted that cholinergic activity is potentiated and increased ACh levels may reflect an inflammatory status in patients with MG.

### Cholinergic components in PBMCs

**Fig. 4:**
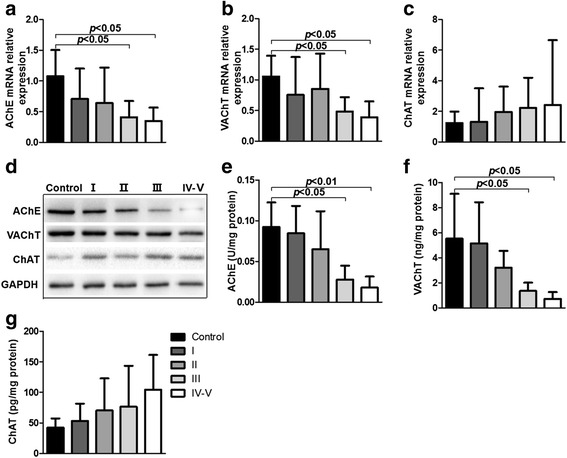
Cholinergic components change in MG patients versus controls. **a** The expression of AChE mRNA was significantly declined in MG patients, especially class III-V, compared with controls (*p* = 0.0130 by Kruskal-Wallis). **b** The VAChT mRNA expression also decreased in MG patients (*p* = 0.0091 by Kruskal-Wallis). **c** There was no obvious change about the ChAT mRNA expression (*p* = 0.2175 by Kruskal-Wallis). **d** Western blots were used to assess levels of the corresponding proteins showing a similar tendency. **e** AChE protein expression in PBMCs detected by ELISA was significantly decreased in class III and class IV-V groups compared with control (*p* < 0.05, *p* < 0.01, respectively). The data were presented as U of AChE per mg protein. **f** VAChT expression was also reduced, especially in class III-V group (*p* < 0.05), compared with control. The data were presented as ng of VAChT per mg protein. **g** The level of ChAT in PBMCs was not significantly different among these groups (*p* = 0.3271). The data were presented as pg of ChAT per mg protein. (**a-c**; *n* = 10 in the control, class I, class II and class III groups, *n* = 6 in the class IV-V group) (**d-g**; *n* = 5 in each group)

When we further explored discrepancies of cholinergic components in PBMCs from MG patients and controls, the relative expression of intracellular AChE and VAChT mRNA had decreased significantly in class III-V patients (*p* = 0.0130, *p* = 0.0091, respectively) (Fig. 4a-b). Yet we did not observe a statistically significant increase in ChAT mRNA expression (*p* = 0.2175) (Fig. 4c). These mRNA levels were similar to the corresponding proteins assessed by Western blotting (Fig. 4d). In addition, the levels of these three proteins in PBMCs were also measured by ELISA. The protein concentrations conformed to the show in Western blotting (Fig. 4e-g). These data thus indicate that the cholinergic activity was changed and the higher ACh levels observed in MG patients perhaps resulted from lower AChE expression.

## Discussion

Our principal findings in this study are that ACh levels increase prominently in PBMCs from patients with MG exceeding those from healthy individuals and ACh likely correlates with the severity of MG. The up-regulated ACh may enhance the lymphocytes’ ability to counteract inflammation via cholinergic signaling to restore homeostasis. Moreover, we observed elevations of circulating anti-MuSK Ab concentrations in MG patients with more serious clinical symptoms, which was accordance with a previous study [29]. Since non-neuronal ACh play an important role in immune regulation, an outcome that likely designates ACh as a possible homeostatic marker induced by the inflammatory reaction in MG.

Experimental evidence indicates that non-neuronal ACh profoundly modulates the innate immune system [7, 31] by binding to nicotinic AChRs such as those that inhibit cytokine release, ameliorate tissue injury and attenuate cytokine-mediated effects in experimental sepsis, endotoxemia and other inflammatory syndromes [8, 32, 33]. On the other hand, the cholinergic anti-inflammatory pathway may be activated by an inflammatory response [10]. Since the roles of PBMC-derived ACh in MG were not previously studied, our study may help to explore the mechanism of MG from a different aspect. Indeed, PBMC-derived ACh levels were significantly higher in MG patients compared with those in healthy controls, particularly higher in class III-V patients according to the MGFA clinical classification. These differences may be caused by the extent of disease, since anti-inflammatory responses are usually determined by the magnitude of the inflammatory response [10]. Under inflammatory conditions in MG, the vagus nerve may be activated by pro-inflammatory cytokines, which could potentiate ACh production by splenic lymphocytes. Therefore, increasing ACh levels in PBMCs likely reflects MG-induced cholinergic hyperexcitation, which potentially serves as endogenous protection from excessive inflammatory reactions. Activation of ACh, therefore, represents a promising therapeutic strategy to improve cholinergic anti-inflammatory function and combat inflammation.

In humans, accumulating evidence indicates that AChE is directly or indirectly involved in the regulation of inflammatory responses or functions as a predictor of inflammation, usually correlated with multiple inflammatory markers, such as those in patients with inflammatory bowel disease [34] or stroke [35]. Presently, AChE inhibitors are used to interfere with the breakdown of ACh and impact the cholinergic system, such as in MG patients. In this current study, AChE expression was significantly lower in patients with MG than in their healthy counterparts, indicating a possible role of ACh content and AChE in regulating inflammation in MG patients.

To explore the potential relevance of ACh levels in the regulation of inflammation in MG, we further conducted a correlation analysis. Surprisingly, we found that ACh levels correlated positively with QMG scores as well as anti-MuSK Ab titers of MG patients. Actually, anti-MuSK-positive patients have a higher frequency of severe clinical symptoms [29]. As previously reported, quantities of anti-MuSK antibodies increased along with MG severity according to the MGFA clinical classification, and correlated with QMG scores in our cohort. As also demonstrated here, the more severe states of disease paralleled the higher PBMC-derived ACh concentrations. We then found that the trend of PBMC-derived ACh content and QMG scores declined in patients after treatment with immunotherapy, which maybe result from the declined cholinergic excitation when patients were in a relatively stable state after treatment. On the other hand, the immunotherapy may influenced the synthesis, storage and degradation of ACh. We therefore speculate that an endogenous ACh-mediated anti-inflammatory pathway may be launched in an attempt to restore homeostasis in MG patients. However, the fact that anti-MuSK Ab levels remained high suggests that, despite higher levels of ACh, this pathway is not sufficient to reverse disease pathology, possibly due to their limited number [19].

Some limitations of our study must be addressed. First, studies *in vivo* and *in vitro* are still necessary to determine the relationships among all these components. Second, the changes of ACh might result from other factors that influence the cholinergic system, such as infection, age or emotion. Further studies should determine their contributions, if any.

## Conclusions

Accumulating evidence suggests that non-neuronal ACh has immunomodulatory effects. Interestingly, we found that elevations in PBMC-derived ACh levels accompanied increasingly severe MG, which may be induced by inflammatory reflex and depend on the magnitude of the inflammatory response. Intervention in cholinergic activity may change the course of this disease. The decline of disease severity after treatment with immunotherapy indicates that ACh levels may be valuable for monitoring the progression or treatment efficacy of MG, which larger and well- defined cohort studies should confirm.

## Additional file

Additional file 1 Detection stability of the ACh levels in PBMCs. a The ACh levels were not changed in PBMCs after pre-adding eserine, a cholinesterase inhibitor, to the peripheral blood sample (*n* = 4 in each group) (*p* = 0.8857). **b** The levels of ACh in PBMCs were not significantly different between untreated and treated MG patients with pyridostigmine (*n* = 8 in each group) (*p* = 0.9768). (PPTX 117 kb)

## Abbreviations

ACh: Acetylcholine
AChE: Acetylcholinesterase
AChR: Acetylcholine receptor
ChAT: Choline acetyltransferase
ChT1: High-affinity choline transporter
MG: Myasthenia gravis
MGFA: Myasthenia Gravis Foundation of America
MuSK: Muscle-specific tyrosine kinase
QMG: Quantitative MG
UPLC-MS/MS: Ultra-performance liquid chromatography-tandem mass spectrometry
VAChT: Vesicular acetylcholine transporter
