# Supplementary material for: Non-neuronal cholinergic activity is potentiated in myasthenia gravis
Source: BMC Neurol. 2017 Feb 8;17:28. doi: 10.1186/s12883-016-0772-3 (PMC5299729; doi:10.1186/s12883-016-0772-3)
Supplement: Additional file 1 — Detection stability of the ACh levels in PBMCs. a The ACh levels were not changed in PBMCs after pre-adding eserine, a cholinesterase inhibitor, to the peripheral blood sample (n = 4 in each group) (p = 0.8857). b The levels of ACh in PBMCs were not significantly different between untreated and treated MG patients with pyridostigmine (n = 8 in each group) (p = 0.9768). (PPTX 117 kb) [file 12883_2016_772_MOESM1_ESM.pptx]

## Slide 1
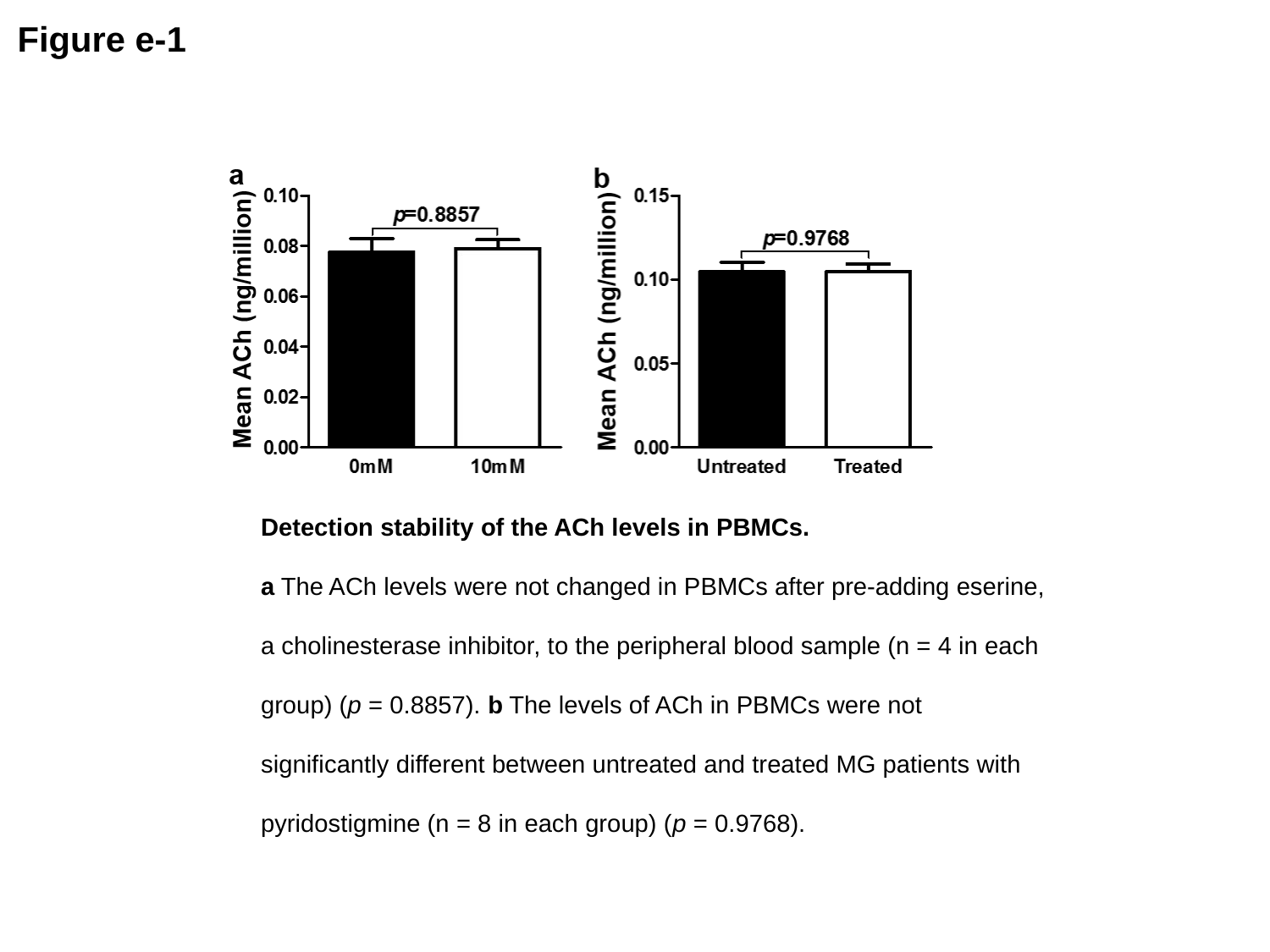

Figure e-1
Detection stability of the ACh levels in PBMCs.
a The ACh levels were not changed in PBMCs after pre-adding eserine, a cholinesterase inhibitor, to the peripheral blood sample (n = 4 in each group) (p = 0.8857). b The levels of ACh in PBMCs were not significantly different between untreated and treated MG patients with pyridostigmine (n = 8 in each group) (p = 0.9768).
